# The evolution of bacterial genome assemblies - where do we need to go next?

**DOI:** 10.20517/mrr.2022.02

**Published:** 2022-04-12

**Authors:** Eric Altermann, Halina E. Tegetmeyer, Ryan M. Chanyi

**Affiliations:** ^1^AgResearch Ltd., Private Bag 11008, Palmerston North 4410, New Zealand.; ^2^Riddet Institute, Massey University, Private Bag 11222, Palmerston North 4442, New Zealand.; ^3^Center for Biotechnology, Bielefeld University, Universitaetsstrasse 27, Bielefeld 33615, Germany.; ^4^Massey University, School of Veterinary Science, Palmerston North 4100, New Zealand.

**Keywords:** Genome assembly, next-generation genome sequencing, single-cell sequencing, assembly, genome variations, artificial intelligence, data formats

## Abstract

Genome sequencing has fundamentally changed our ability to decipher and understand the genetic blueprint of life and how it changes over time in response to environmental and evolutionary pressures. The pace of sequencing is still increasing in response to advances in technologies, paving the way from sequenced genes to genomes to metagenomes to metagenome-assembled genomes (MAGs). Our ability to interrogate increasingly complex microbial communities through metagenomes and MAGs is opening up a tantalizing future where we may be able to delve deeper into the mechanisms and genetic responses emerging over time. In the near future, we will be able to detect MAG assembly variations within strains originating from diverging sub-populations, and one of the emerging challenges will be to capture these variations in a biologically relevant way. Here, we present a brief overview of sequencing technologies and the current state of metagenome assemblies to suggest the need to develop new data formats that can capture the genetic variations within strains and communities, which previously remained invisible due to sequencing technology limitations.

## CURRENT SEQUENCING TECHNOLOGIES

Our ability to decipher DNA, the building blocks that define the state and evolution of life, has fundamentally changed our understanding and interaction with living beings.

It is becoming commonplace in many laboratories to perform whole-genome sequencing and create a draft genome assembly as the first step of working with a new organism. It was not very long ago where that idea would have been considered cost-prohibitive and unrealistic. Genome sequencing technologies have come a long way in a surprisingly short time. Advances in sequencing technologies and computational pipelines for genomic data reconstruction have made these experiments tenable for non-experts and have allowed scientists to pose questions that were not even imaginable a few years ago. Now in the era of “big data”, each new advancement is pushed along two fronts. The first one is the sequencing technologies themselves in a never-ending battle for more data with increased accuracy using poorer samples. The second is the computational pipelines to handle this information to get a better understanding of what the data is telling us. 

Although Sanger receives much of the credit for modern DNA-sequencing, Wu and Taylor^[1]^ were one of the first to report the determination of a DNA sequence; however, many of the techniques used were adapted from previous work from Sanger^[2-4]^ and Holley *et al.*^[5]^. Further DNA-sequencing approaches were developed^[6-8]^; however, these tended to be rather laborious until Sanger published the now famous “chain-terminating” inhibitors approach^[9]^. This technique has gone through many iterations and has been greatly improved, and the core technology remains widely known as “Sanger Sequencing”.

Throughout the 1980s, shot-gun sequencing approaches accelerated the pace of DNA sequencing^[10]^. It was realized that fragments of a genome could be randomly digested and ligated into bacterial vectors. These vectors could now be readily sequenced thanks to Sanger-based sequencing approaches, and regions of overlap were used to place the DNA sequences for allowing the sequence of whole genomes to be determined. The idea that whole genomes could now be sequenced spurred The Human Genome Project (HGP).

The impact Sanger sequencing has had on the field cannot be understated, but, as with any scientific discovery, there is always a push for more. Sanger sequencing builds from a single primer present in the sample. Therefore, if more than one primer is present, or if the sample contains another DNA fragment that the primer can bind to, the correct sequence of interest cannot be determined. Also, only a single strand can be sequenced at one time. With many advances in technology and sample automation strategies^[10-12]^, this technique soon reached its peak performance. However, it should be noted that Sanger sequencing is one of the most widely used tools in laboratories today in terms of sequence verification and molecular biology applications but is less practical in the era of omics data.

**Figure 1:**
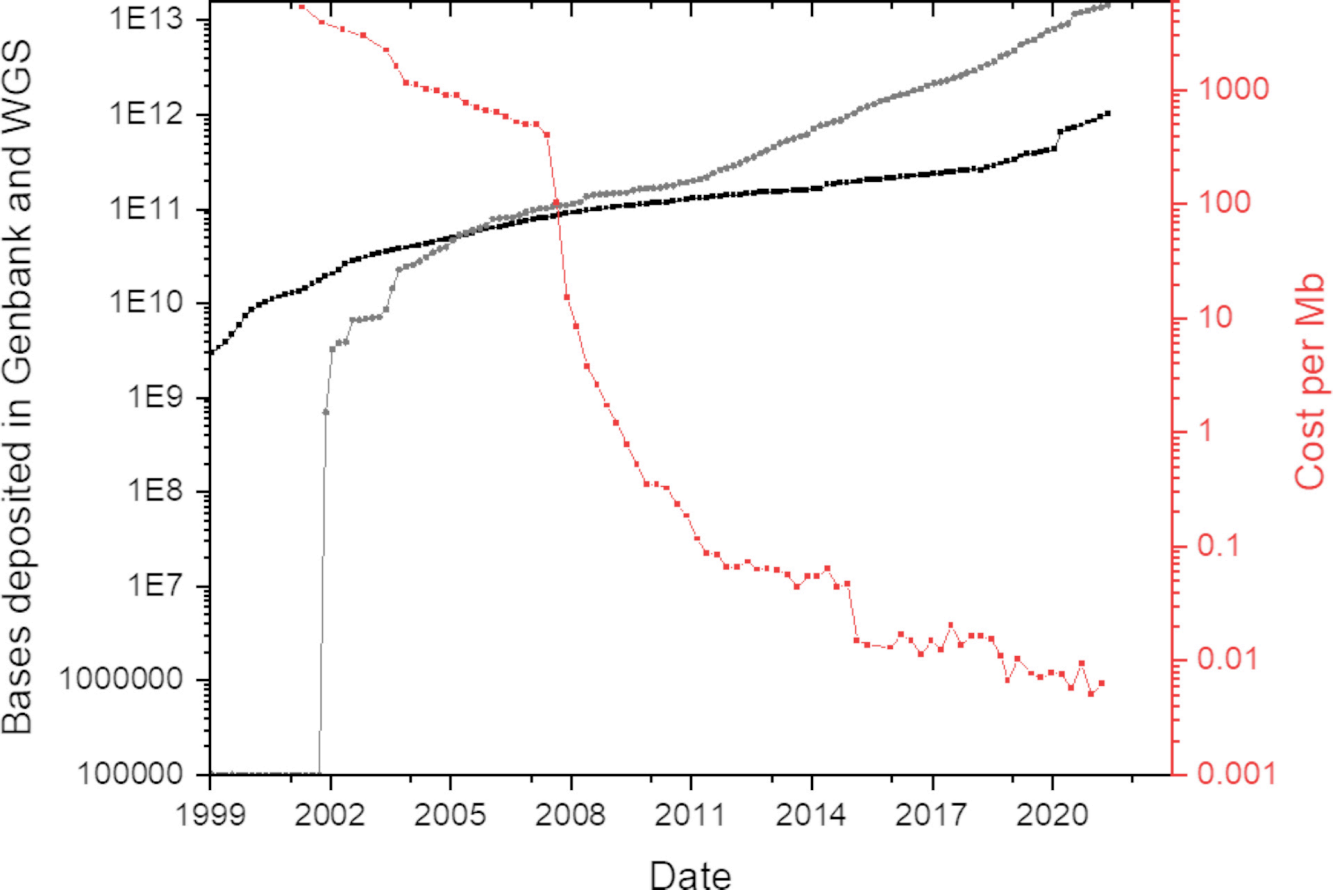
Cost versus sequences deposited. Sources: genomes.gov & NCBI. Amount of sequence deposited doubles roughly every 18 months and this is likely to accelerate as long-read technologies further mature. Red: Cost to sequence a Mbp of DNA; Black: individual sequences directly deposited into Genbank; Grey: WGS sequence data deposited (April 2002); WGS: whole-genome sequencing.

It is estimated that the cost associated with the HGP was $2.7 billion USD. Scientists recognized the value of this technology, and companies understood that revenue could be generated by decreasing the costs to make it more accessible. Figure 1 highlights the average cost of sequencing (per MB) as compared to the amount of sequencing data being deposited into NCBI. As we see new technologies emerging, the cost is significantly decreasing. These new technologies are now collectively referred to as next-generation sequencing (NGS) technologies and can be further characterized into short-read (pyrosequencing, Ion Torrent, Illumina), long-read (SMRT: PacBio and Nanopore), and next-gen (next step in sequence evolution). 

### Short-read NGS-technologies

Broadly speaking, the advances behind most NGS technologies rely on the detection of the signal when a nucleotide is added to the elongating strand, known as sequencing by synthesis (SBS). Short-read sequencing technologies, like Illumina, typically only extend up to 250 nt from a single strand; however, they can sequence thousands (minimally) of strands simultaneously (Massive Parallel Sequencing). To enhance the signal, either solid phase bridge amplification^[13]^ or emulsion PCR is used for Illumina and Ion Torrent sequencing technologies, respectively. Although two slightly different approaches, the result is the same, whereby a single DNA strand is PCR-amplified while adhered to a surface (or bead) to create a small zone of identical DNA molecules, known as a polony. SBS approaches, such as Pyrosequencing (also known as 454), Ion Torrent and Illumina, use very sensitive measurement techniques to identify the added nucleotide. A single surface, chip or bead, can be coated with thousands of polonies which would duplicate the genetic information in the sample many times over. If beads are used, these can be separated into microscopic wells such that a single bead containing a single DNA sequence resides in its own isolated well. 

Pyrosequencing was the first NGS platform of note. This platform was based on the enzymatic detection of the pyrophosphate that is released when a nucleotide is added, causing the release of light as a signal^[14]^. The optics cover the entire chip being sequenced and can simultaneously sequence thousands of polonies one nucleotide at a time. Although this technology provided a step-change in sequencing techniques early in NGS development, it ran into issues of poor optics and is known to deal poorly with DNA strands containing homopolymer stretches beyond 5 or 6 bp. Ion Torrent functions based on the same principles; however, instead of optics-based determination of bases, Ion Torrent measures the release of hydrogen across an ion semiconducting surface. Similar to previous methodologies, the Ion Torrent platform ran into serious issues if homopolymers were present, over-calling short homopolymers and under-calling longer homopolymers^[15]^. Although improvements have been made^[16]^, advances in other NGS technologies soon overtook the pyrosequencing and Ion Torrent platforms^[17]^. Although Ion Torrent still finds use today due to its speed and read length (> 600 bp), pyrosequencing was discontinued in 2013.

The most popular SBS short-read platform is Illumina. The general principle behind the technology is similar to previous NGS platforms. Fluorescent-based nucleotides are used with the fluorophore located on the 3 end blocking DNA polymerase from adding more than one base. Therefore, only a single base can be added to a strand per cycle, allowing for accurate determination without errors due to homopolymers. Illumina is currently the preferred choice for short-read sequencing due to the low DNA concentrations required and a low error rate of base calling.

### Long-read NGS-technologies

Long-read technologies delivered another step-change by introducing much longer sequence data ranging from 10 kb to 30 kb; however, there has been a report of a single read that potentially spanned 2.27 Mbp^[18]^. The PacBio sequencing platform^[19]^ uses single-molecule real-time (SMRT) sequencing. The fragmented DNA has adapters ligated onto each end which circularize individual strands. Millions of these fragments are immobilized in the small wells of an SMRT chip. Similar to Sanger and Illumina sequencing methods, PacBio employs fluorophore-labelled nucleotides for base calling. PacBio sequencing produces very large read lengths (> 50 kb) that repeat due to the circularity of the template. A recognized problem with this technology is the high error rate of 10% to 15%^[20]^ for each base call, but these errors are random. Therefore, due to the circularity of the strand and the fact it is sequenced many folds over, these errors are corrected using bioinformatic tools^[21]^.

Oxford Nanopore Technologies (ONT) is the other leading long-read platform but uses an entirely different approach. ONT uses pores in a lipid membrane that has an electrical potential, with the exterior being more negatively charged. This promotes the DNA molecule to pass through the pore, with the ionic current disrupted proportionally to each respective nucleotide. By reading these characteristic changes in ionic current, the sequence of the DNA molecule can be determined. A miniaturized chip containing hundreds to thousands of these pores is used to sequence a DNA library. Each pore can be used to sequence multiple DNA fragments, as it becomes free and re-usable once a fragment has passed through it. Similar to PacBio, ONT suffers from an overall error rate of ~10%, but this can be significantly reduced with error-correction tools to about 3%^[22]^. The major advantage of using the ONT platform is that it requires minimal reagents, preparation and can be portable. For example, the MinIon unit is smaller than the average cell phone and can be plugged into a laptop to run^[23]^. Two major issues are associated with ONT: first, the DNA molecule is passing through the pore very quickly, and second, many bases are in a pore at one time; both factors together can obscure the electric signal, limiting the accuracy of base calling. 

Stratos Genomics (purchased by Roche Diagnostics) has developed a similar approach that expands upon the ONT design. Termed “Sequencing by Expansion” (SBX), this technique links each nucleotide base with a specific signalling molecule (“Xpandomer”) that is covalently bound to the adjacent Xpandomers. Therefore, each Xpandomer can be used as a proxy for the nucleotide it represents. The DNA molecule is degraded, which allows the Xpandomers to spread out but still represent the original DNA sequence. These molecules are passed through a nanopore, and as each Xpandomer passes through, the electrical resistance changes proportional to the molecule passing through. Since Xpandomers are larger and spaced out further than the corresponding nucleotides would be in a DNA strand, the signal is easier to read. Unfortunately, at the time of writing, SBX-technology was not yet commercially available and is still in the development phase. Therefore, it is not possible to comment on whether this technique would improve the error rate of ONT, but if successful, it would constitute an improvement of long-read technologies.

## THE ASSEMBLY OF METAGENOME ASSEMBLED GENOMES - CONCEPTS, STRATEGIES AND CHALLENGES

A very promising aspect of high throughput sequencing in the field of microbiology was the possibility to study microbial communities directly on the genome level. The thousands to millions of generated sequence reads from just one NGS run could cover hundreds of microbial genomes. This increase in sequencing coverage compared to Sanger-based sequencing made it possible to obtain direct insights into the DNA composition of samples containing a multitude of organisms. A sequence data set obtained from such samples is called a metagenome. Metagenomes were studied both directly on sequence read level, or an assembly of the short sequence reads into longer contigs was attempted to obtain complete gene sequences or operons from the data. Metagenome analysis became a powerful strategy to study not (yet) culturable microorganisms^[24]^. It was now possible to obtain information about so far unknown taxa and the potential functional properties of microbial genomes directly recovered from natural habitats^[25,26]^.

The sequencing coverage per NGS run continued to increase, and assembly tools were adjusted to fit metagenomic data, which eventually enabled the recovery of entire microbial genomes from sequence data of complex microbial communities. As shotgun sequencing evolved from elaborate and resource-consuming procedures to be much more time and cost-effective, so did the recovery of metagenome-assembled genomes (MAGs) (metagenome-assembled genomes) from the generated sequence data.

The first published MAGs were obtained from shotgun Sanger sequencing of a low complexity metagenome^[27]^, yet as NGS technologies advanced to be widely available, metagenomes were then mainly generated via Solexa/Illumina, 454 or Ion Torrent sequencing. However, the assembly of MAGs from these relatively short read data was more challenging than the assembly of longer reads. In particular, the short reads made it difficult or impossible to assemble repetitive sequences or regions encoding highly conserved genes with several copies present in the genome, such as ribosomal genes^[28,29]^. Sequencing strategies such as mate pair sequencing and artificial long reads mitigated some of these drawbacks, but it made the sample processing and sequence generation, as well as the post-sequencing data processing steps, more elaborate and time-consuming.

Therefore, initially, a “MAG” was not a complete closed genome, but rather an assembly of several (up to hundreds of) contigs that were assigned to a taxon, often described as an operational taxonomic unit (OTU). Assigning contigs to an OTU or MAG usually involved a binning step, based on factors such as k-mer frequency, the abundance of assembled genes, alignments to reference databases, or a combination of several strategies^[30-32]^. The quality and completeness of such MAGs were then determined by the presence of genes encoding RNAs and universal single-copy genes^[33,34]^.

In order to obtain high-quality MAGs or even closed genomes from metagenomic data, long read sequencing technology platforms described above lead to substantially improved assembly results. Parallel to this, software tools optimized for metagenome assembly and binning were developed, making it possible to assemble single contig MAGs from complex metagenomes^[35,36]^.

### Metagenome assembly 

Metagenome assembly in general usually comes with the dilemma of deciding whether the emphasis lies in assembling as many genomic regions as possible or whether fewer but more accurate, high-quality sequences should be obtained by setting more stringent assembly parameters, which usually results in a lower percentage of assembled data. A clear understanding of which strategy should best be followed to answer a particular research question is crucial, as it influences the optimal choice of sample processing, sequencing technology, and assembly procedure^[37]^. If the focus lies on a broad overview of the functional potential captured by a complex metagenome, deep short-read sequencing and less stringent assembly parameters will likely yield more of the required information than long-read sequencing with lower coverage. The latter will be the better strategy if the assembly of closed high-quality MAGs of the dominant organisms is the main goal. Furthermore, the generation of complete and accurate MAGs to date still requires a meticulous process of assembly curation and decontamination, which is difficult to automate due to differences in sequence composition between different metagenomic samples^[38]^.

### Assembly tools 

Assembly tools have developed in parallel to the evolution of NGS technologies. Initially, MAGs were assembled using the tools developed for genome assembly from pure culture sequencing, which caused drawbacks regarding contamination of MAGs with non-specific sequences, especially if the sample contained closely related species and/or no particularly dominant taxa. The procedure often resulted in chimeric assemblies as the tools did not inherently differentiate between sequences of several genomic origins. Differences in genome coverage posed another challenge, and the high complexity of the data made a single-genome assembly strategy computationally challenging^[39]^. As metagenome assembly became more common, the existing assembly tools were either adjusted to allow parameter settings for multispecies sequence data^[40]^, or new tools were developed to specifically assemble metagenomic data^[41,42]^. These tools are then often optimized for a particular type of sequence data (long- or short-read data) in order to account for sequence data characteristics, such as paired-end information, and expected rates and types of sequencing errors^[43,44]^.

Further important tools for the generation of high-quality MAGs are binning tools, which allow for sorting assembled contigs into bins, each of which likely constitutes a MAG (or a taxon/OTU). These bins can then be used to further improve the assembly of the MAGs they represent - usually by the re-assembly of all original read sequences assembled into the contigs that were sorted into a particular bin^[36]^. The optimal choice for specific assembly and binning tools depends on the original data set at hand, as there are differences in their performance with regard to species composition and similarity in the original sample^[45]^. Detailed descriptions and comparisons of metagenome assembly and analysis tools can, for example, be found in Yang *et al.*^[44]^ (2021), Anyansi *et al.*^[46]^ (2020), and in Ayling *et al.*^[45] ^(2019).

One of the main challenges of metagenome assembly is the distinction between sequencing errors and sequence diversity in the DNA sample. Depending on the sequencing technology, insertion-deletion errors (indels) or substitution errors (SNPs) need to be accounted for in order to accurately assess the genetic diversity of the sample. Long-read sequencing is more prone to indels, which can cause truncated open reading frame (ORF) calling in an assembled MAG due to artificial frameshifts. Polishing tools for long reads and long-read assemblies improve their error rate^[47]^. Another strategy is to combine the assembly of indel-prone long reads with short-read data (Illumina) that contain SNPs, rather than indels. These so-called hybrid assemblies allow for long read error correction using short-read data, and thus typically result in higher quality MAGs, despite a relatively low sequencing coverage^[48]^. As both chemistry and base calling processes for long-read sequencing continue to improve, MAG assemblies from long-read data only are becoming more accurate^[35,49]^.

A drawback of long-read sequencing is the amount of high molecular weight (HMW) DNA needed for the sequencing library preparation. Contrary to most Illumina sequencing protocols, there is no or only little DNA amplification involved in the library preparation and sequencing process of long-read sequencing platforms. This makes it difficult for small samples to be sequenced with long-read platforms. Small sample DNA amplification using multiple displacement amplification (MDA) prior to library preparation is possible; however, it can lead to highly uneven genome coverage due to amplification bias^[50]^. Furthermore, due to the highly branched nature of the DNA after MDA, the library inserts will not be as long as they usually are with directly extracted HMW DNA^[51]^, which might limit the assembly of very long contigs and require higher sequencing depths.

Computational studies on microbial evolution^[52]^ have been instrumental in developing tools to examine the genomic variation of closely related organisms to identify SNPs and indels and distinguish them from sequencing errors. These approaches relied upon reference-based alignments of the sequencing data. Currently, these strategies are not sufficient to distinguish between highly similar genomes in a metagenomic data set for which no reference genomes are present, and which therefore can only be assembled via a non-reference-based *de novo* assembly approach.

One major issue still to be addressed is the separation of strains in a *de novo *microbiome assembly. Initially, with only short-read data at hand, strain separation was attempted via haplotype assembly strategies. It required a high degree of sequencing coverage for the assembly algorithms to reliably assign the reads originating from only slightly different chromosomes. For strains or related species of similar frequency, chimeric assembly occurred when highly similar genomic regions could not be resolved^[46]^. Most of the currently published MAGs are likely to be at least partially chimeric consensus sequences of several strains, or even closely related species. Strain separation within a bin of *de novo* assembled contigs assigned to a taxon or MAG can, for example, be attempted by frequency assessment of sequence variations in the assembled reads^[53]^, or by searching for similar frequencies of nucleotide variants detected in conserved single-copy genes^[54]^. The outcome of these procedures relies on the quality of the assembled MAG contigs, which stresses the need for a thorough overall concept for the sequence data generation and processing in order to obtain meaningful results.

## FUTURE CHALLENGES

Matured short- and long-read sequencing technologies have opened the doors for deep shotgun metagenome sequencing and, more recently, enabled the assembly of MAGs at larger scales. New scientific horizons have begun to emerge. With increasing sequencing depth and read length, sufficient coverage of rare members of microbial communities will become possible, enabling the creation of high-quality MAGs that will help to ascertain the role and contribution of these rare members to the overall community fitness. As described above, the quality of MAGs is ever-increasing, and we anticipate high-quality draft and closed MAGs to emerge routinely over the next five years. As these (complete) MAGs become increasingly available not only for major clades within a microbial community but also for increasingly rare members, we will also be able to begin capturing strain variants within a population, including their respective variant frequencies. It is a long established fact that even in homogeneous bacterial cultures originating from a single cell, sub-populations with genetic variations emerge over time. The same is true in natural microbiomes where ongoing and sometimes rapid evolution creates a reservoir of sub-populations with genetic and possibly phenotypic variations that are not negatively affecting their respective survival. These silent sub-populations enable rapid responses and adaptation to change environmental pressures and are a key driver to the survival of a given microbial species and strains. Without these sub-populations, the microbiome’s ability to quickly respond and adapt to any environmental pressures would likely be severely compromised. But these sub-populations on strain level have so far eluded recognition and (full) characterization, despite their importance for ongoing bacterial genomic evolution and lifestyle adaptations, impact on personalized medicines, and biotechnological and pharmaceutical fermentation^[55,56]^. 

Maturing long-read and emerging next-gen^[57]^ sequencing technologies, that will again deliver longer read lengths at reduced error rates compared to current long-read technologies, will deliver on the promise of deep MAGs that will detail genomic variation in unprecedented detail. However, these new frontiers come with formidable and interlinked challenges. 

The growing volume of sequence information originating from the deep community and single-cell sequencing will necessitate reliable and automatic genome assemblies paired with high throughput detailed genome annotations. Although advances in long-read technologies are predicted to generate reads that could span > 100 kb, making it easier to assemble closed genomes, this will be offset by the amount of sequence generated with the maturation of single-cell genome sequencing. Reliable and fully automated structurally correct assembly of genomes and genome closure will require more advanced use of artificial intelligence (AI) and machine learning. AI systems will need to be able to self-train structurally correct assemblies based on existing reference assemblies and deep machine learning algorithms capable of recognizing and reconciling structural genomic variations. As new assembly and annotation algorithms emerge, AI systems will autonomously test, evaluate and, if deemed suitable, incorporate these into their existing repertoire of tools. Ongoing improvement and optimization will likely require cloud-based systems capable of providing required computational resources. For end-users or confidential data, static snapshots will be spawned on a regular basis that can be installed and executed on less powerful, local workstations. It is hard to predict how quickly such systems will evolve and whether they will be deployed on conventional silicon or make use of quantum computers capable of solving more complex networks. While the prospect of quantum computing is tantalizing, its use is currently limited to very specific types of problems and programming languages, and concepts have barely begun to emerge, allowing for broader fields of applications^[58]^. However, forecasts predict a much wider use of quantum computers over the next decade^[59]^, and their application in next-generation genome spaces, defined as the collective of (accessible) draft and complete genomes, is highly likely.

The second challenge is directly enabled by the emerging ability to investigate populations in detail on the genome level and thus capture genetic variances on a strain level, initially as a static snapshot and eventually including longitudinal changes in response to environmental changes. How can this variation be captured and recognized in a (human) readable format?

Historically and at present, genome assemblies of microbial populations are assembled based on determining a single consensus sequence. Sub-populations on the strain level that feature genomic variations are discarded. However, with the emergence of single-cell sequencing and the availability of high-quality draft and closed genomes assembled from communities and microbiomes, opportunities arise to investigate the contribution of such sub-populations to the rate of evolution, the fitness of a population, and their ability to respond to environmental challenges and pressures. Such a deep understanding of genomic variation below the strain level would also enhance our ability to predict the impact on health and well-being of humans and animals as we begin to better understand how beneficial and pathogenic microbes evolve, how genomic hotspots with rapid evolution emerge and contribute to phenotypic change, and at what frequencies such sub-populations are maintained or begin to disappear. 

To begin addressing this emerging opportunity, we first need to consider thresholds upon which base call errors can be distinguished from the presence of subpopulations. This will likely be sequence technology-specific and, from today’s perspective, comprises a statistical approach combining known error rates with sequence depth coverage for at least one long-read and one short-read sequencing chemistry. This implies a minimum coverage needed for each genome position that enables not only the establishment of an error rate threshold but also allows for sufficient information to detect sub-populations. 

Identifying sequence reads from metagenomes or (assembled) genomes from single-cell sequencing that belong within the same strain genome space is the next step in the evolution of genome assemblers. Here, the definition of a strain genome space requires some consideration and may need to be flexible enough to accommodate different evolutionary strategies deployed by different microbes. Perhaps a first model could consider the presence and absence of SNPs. In such a model, only minor changes between individual sequence reads and/or assembled genomes are permitted. The capture of such variations can then be readily realized by representing each genome position by five possible states: A, T, G, C, and Gap. Each state can be attributed a normalized distribution value, ranging from 0 to 100% of the total sequence coverage at this genome position. Consequently, the consensus genome for a strain genome space would appear to be slightly larger than individual sub-population specific genomes to accurately reflect insertion events present in individual sub-population genomes. 

**Figure 2:**
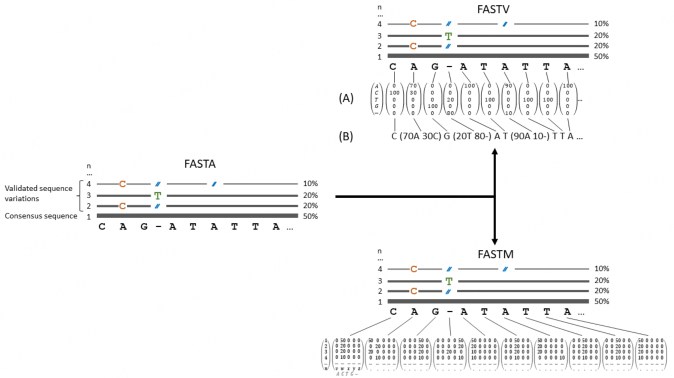
Potential FASTA successor formats FASTV and FASTM. For FASTV, each genome position is depicted either by a matrix “()” (FASTV A) or a human readable notation (FASTV B), where a single genome location comprises the normalised distribution value of one of the four bases or a gap, ignoring bases that do not occur at this position. In FASTV, the frequency of nucleotides per position is captured but not their respective occurrences across subpopulations. For FASTM, a two-dimensional matrix enables capturing not only the relative distribution of nucleotide variations for every genome position, but the matrix further enables the tracking of individual subpopulations.

Current methods to record sequence information and annotation cannot effectively capture this genomic variation. Data formats such as FASTQ^[60]^ capture the quality of a base call at a given position with a sequence read, while FASTA represents consensus sequences without any quality qualifiers. Similarly, file formats such as Genbank can provide information on the first higher abstraction level - gene models and annotation - but cannot represent genomic sequence variation. Sub-population variation on strain levels could be represented by a variation of the FASTA format, as described above. A potential new FASTA file format, tentatively named “FASTV”, is shown in Figure 2 (FASTV). Each genome position is represented by either a one-dimensional matrix (FASTV A) or a more human-readable notation (FASTV B). The FASTV format captures positional variations but does not differentiate between individual subpopulations. This FASTV format could then be seamlessly integrated into a Genbank file format analog. Here, the way gene models (and hence annotation) are described also needs to be modified. One approach could be to allow multiple ORFs per locus tag qualifier, again assigning a normalized frequency to each locus tag associated with a given subpopulation.

The main drawback of such a simplistic expansion of available file formats is that the sub-population structure is lost, and genetic changes within individual sub-population genomes cannot be traced.

**Figure 3:**
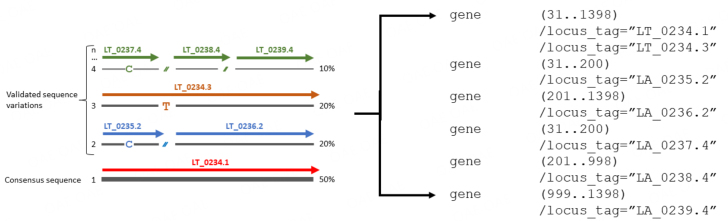
Possible translation of sub-population sequence variations into a Genbank-like format. Sequence variations that do not change the gene model retain the same locus tag (e.g. LT_0234) but change the appendix to refer to the respective subpopulation. Where gene model changes occurs, e.g., sub-populations 2 and 4, new locus tags are issued along with the respective sup-population appendix.

Possible evolution of the FASTV file format that enables accurate sub-genome tracking could be a two-dimensional matrix file format where one dimension is reserved for genome position and the second dimension recognizes each identified sub-population. A tentative file format, dubbed FASTM, is suggested in Figure 2 (FASTM), where each matrix presents a single genome position. With each matrix, the first column represents identified sub-populations while the remaining columns depict the normalized frequency of each possible state in the order A, C, T, G, and - (gap). Therefore, the FASTM format is a representation of a sequence data set, with the sequence reads already aligned and assembled. Sequencing errors have already been checked and removed using the appropriate tools/statistics etc., assuming sufficient sequence coverage. The FASTM matrix then captures, with the required significance, different variants within a single strain or different strains within one species in their respective abundances. Similar to the FASTV format, FASTM matrices could be incorporated into Genbank-like file formats and sub-population-specific gene models indicated through locus-tag qualifiers [Figure 3]. 

These new concepts for recording and analyzing genetic variations on sub-strain populations are far from perfect, and we suggest a concerted effort of the science community to further refine these file format suggestions to be prepared for these upcoming challenges.

We also acknowledge the limitations of these new concepts and formats. While we believe that, in principle, they will be capable of accurately reflecting nucleotide changes, insertion/deletion events, and horizontal gene transfer events of any size, they are not suitable to capture genome rearrangements of any size. It may be a worthwhile discussion to define at which point observed genome rearrangements (or the loss and acquisition of loci) still represent a sub-population of the same strain and when these changes warrant the definition of a new reference strain. If genome rearrangements are allowable features within a strain sub-population, the suggested FASTV and FASTM format will no longer be suitable and should be replaced. Another limitation is the relevance these new formats will carry with respect to the quality of meta-genome assemblies. Ideally, these formats are applied to closed genomes or high-quality draft assemblies comprising long scaffolds or contigs. For assemblies of lower quality and thereby decreasing scaffold/contig lengths, it will be more difficult to confidently assign metagenome reads and detected sequence variants to individual assemblies and contigs therein and scaffolds/contigs to genomes. With ongoing advances in sequencing technologies and assembly tools, this limitation may only be a temporary one until high-quality assemblies and closed genomes have become the norm.

## CONCLUSION

Ongoing advances in both sequencing technologies and assembly tools are paving the way for a deeper and more detailed understanding (and tracing) of evolutionary changes in the genetic blueprint of individual strains. Current data formats are not fully suitable for managing these new levels of detail, and here we have provided two initial formats that may begin addressing this challenge. We hope that these suggested formats may serve as a starting point for a larger science community discussion on how to best capture sub-population dynamics on a genome assembly level and identify which advances will be required in assemblers and annotation pipelines to leverage this new level of detail.

Looking further into the future, such new formats alongside advances in machine-learning driven assemblers may also serve to describe longitudinal changes in populations and their respective assembled MAGs, perhaps for the first time allowing to follow directly emerging genetic adaptation and population changes on a sub-strain level. 

## DECLARATIONS

### Copyright

© The Author(s) 2022.
